# Heresy or Truth?

**Published:** 1921-01-29

**Authors:** 


### CLEARING THE GROUND.

### Heresy or Truth?

May I be permitted to discuss a heresy?

A heresy is not necessarily false. It is simply a doctrine held by a minority. It used to be a heresy to regard women as eligible to vote at Parliamentary elections. It used to be something worse to regard women as justified in smoking, and if one goes a little further back, the top of an omnibus or a hansom cab were strictly forbidden. It would be difficult to enumerate the number of heresies that have gone under in the life of the present generation. Hence my claim to discuss one that survives.

A Sound Unorthodox Doctrine.

It is a sound orthodox doctrine in the nursing world that nurses should not, or, to be accurate, cannot, be effectually represented in their organisations by any save doctors or nurses. An outsider is regarded in select circles in much the same way that Quakers look upon members who "marry out." I am phillistine enough to believe that Quakers ought to "marry out," and likewise that neither nurses nor doctors are necessarily the best representatives that nurses can choose to voice them in public. There are, of course, always brilliant exceptions. I know one or two. It would be invidious to name them, but I hold that they must be exceptions, and for obvious reasons.

Let us consider. If you were electing a nurse or a doctor, what type would you prefer—a highly skilled well-known member of either profession, or one who is obscure? The question has only to be asked : the answer is obvious. Now the business of a doctor or a nurse is in no way calculated to fit him or her for public life, a remark which applies especially to a nurse. Those who have been oft-times present at meetings of nurses testify that although they may be alert, bright faced, intelligently interested, they are almost always silent. Their occupation and their discipline tend to make them so. The atmosphere of the sick room is one of hush, and when nurses go off duty it is not to a debating society that they wend their weary way. Those nurses who shine at public meetings do so despite their training and their environment.

It is quite possible that this idea of nurses representing nurses found its origin in the labour movement. Miners represent miners in Parliament, and farmers choose farmers. But the leaders in these cases graduate in the club-rooms of Oddfellows, Foresters, Druids, and kindred societies. They study the economics of Trade Unions and the laws that govern employer and employee. They learn the rules of debate and the art of heckling, and, naturally, the man who leads may one day find himself nominated to contest a Parliamentary election. From such training and such publicity the majority of women, and certainly the majority of nurses, would shrink.

A Motive of Necessity.

I am not engaged in any academic discussion, nor is my criticism of a cherished tradition of the profession made with any motive other than that of necessity. The constant energy of skilled organisers is absolutely essential to the economic progress of the nurse. And, no matter how people with pious intention may preach the doctrine of non-resistance to nurses, things will go from bad to worse unless there is a vigorous campaign of reform conducted by somebody. To quote the matron of the Royal Free Hospital, she declares that she requires eighteen probationers, and has not one on her waiting-list. The chairman of the meeting where this observation was made, Mr. Bircham, of the Legal Department of the Ministry of Labour, asked, in reply, if the nurses had a forty-eight-hours' week, or were paid overtime. The answer, of course, was in the negative, and the inference is unmistakeable, that the status of the trained nurse must descend if the economic conditions of her employment are not improved.

I venture to think that if the facts are carefully examined it will be generally agreed that, no matter how strongly doctors and nurses may feel on the subject, they themselves are not, because of their occupation, necessarily the most suitable instruments for influencing public opinion. In nominating College election candidates, therefore, perhaps these remarks may be seasonable and useful.

I have always appreciated the wisdom of the founders of the College in making it a rule that anybody, man or woman, whom nurses might choose, should be eligible to represent them, and the present Council contains, I believe, at least two members who are not doctors or nurses.

IERNE.

